# Visual Working Memory of Chinese Characters and Expertise: The Expert’s Memory Advantage Is Based on Long-Term Knowledge of Visual Word Forms

**DOI:** 10.3389/fpsyg.2020.00516

**Published:** 2020-04-17

**Authors:** Hubert D. Zimmer, Benjamin Fischer

**Affiliations:** ^1^Brain & Cognition Unit, Department of Psychology, Saarland University, Saarbrücken, Germany; ^2^International Research Training Group “Adaptive Minds”, Saarland University, Saarbrücken, Germany

**Keywords:** visual working and short-term memory, visual working memory c, expertise, visual working memory precision, Chinese character, visual word form area

## Abstract

People unfamiliar with Chinese characters show poorer visual working memory (VWM) performance for Chinese characters than do literates in Chinese. In a series of experiments, we investigated the reasons for this expertise advantage. Experiments 1 and 2 showed that the advantage of Chinese literates does not transfer to novel material. Experts had similar resolution as novices for material outside of their field of expertise, and the memory of novices and experts did not differ when detecting a big change, e.g., when a character’s color was changed. Memorizing appears to function as a rather abstract representation of word forms because memory for characters’ fonts was poor independently of expertise (Experiment 3), though still visual. Distractors that were highly similar conceptually did not increase memory errors, but visually similar distractors impaired memory (Experiment 4). We hypothesized that literates in Chinese represent characters in VWM as tokens of visual word forms made available by long-term memory. In Experiment 5, we provided novices with visual word form knowledge. Participants subsequently performed a change detection task with trained and novel characters in a functional magnetic resonance experiment. We analyzed set size- and training-dependent effects in the intraparietal sulcus (IPS) and the visual word form area. VWM for trained characters was better than for novel characters. Neural activity increased with set size and at a slower rate for trained than for novel characters. All conditions approached the same maximum, but novel characters reached the maximum at a smaller set size than trained characters. The time course of the bold response depended on set size and knowledge status. Starting from the same initial maximum, neural activity at small set sizes returned to baseline more quickly for trained characters than for novel characters. Additionally, high performers showed generally more neural activity in the IPS than low performers. We conclude that experts’ better performance in working memory (WM) is caused by the availability of visual long-term representations (word form types) that allow a sparse representation of the perceived stimuli and make even small changes big because they cause a type change that is easily detected.

## Introduction

Visual working memory enables the storage of about three to four visual objects for a short period of time (Luck, 2008; Zimmer, 2008; Luck and Vogel, 2013; Mance and Vogel, 2013). It is therefore assumed that VWM has a highly limited capacity, where “capacity” denotes the number of items or features that can on average be remembered. Capacity is commonly estimated by the performance in a so-called change detection task. A study picture displaying a varying number of objects is briefly presented, and after a short interval of about 1 s, a test display is shown. The object(s) in the test display are either the same as those in the study picture or presented with some changes. The participant’s task is to detect these changes. The classical result is that performance is nearly perfect up to a set size of three to four items and subsequently decreases sharply (Luck and Vogel, 1997). When specific assumptions about the decision process are made, the proportion of changes identified correctly can be used to estimate capacity as the number of items that can be represented in working memory (WM). Across many domains, it is around four (Cowan, 2001). This capacity is smaller if the items to be memorized are visually complex (Alvarez and Cavanagh, 2004). For Chinese characters, the capacity of VWM can be as low as one character (Zimmer and Fu, 2008; Sun et al., 2011; Schurgin and Brady, 2019). However, this effect varies strongly with the expertise of the participants. Literate Chinese show better memory for familiar than unfamiliar characters (e.g., Yu et al., 1985; Zhang and Simon, 1985), and experts have a higher capacity for Chinese characters than novices who are inexperienced with this material (Hue and Erickson, 1988; Sun et al., 2011).

The aim of this paper is to investigate the mechanism of this expertise advantage in VWM. In a series of experiments, we will show that knowledge of visual word forms is the key factor. We assume that this allows experts to represent characters as sparse code at a structurally high level. We consider the VWM entry as a token of the word form representation (the character’s orthography) in long-term memory. By this mechanism, the percepts of complex visual characters are reduced to token representations of known items. In change detection tasks, such representations allow an item change to be detected easily because the change causes a shift to a different “visual category” (a different character), and it does so even if the change is perceptually small. Oberauer and Eichenberger (2013) speculated that “the unit of VWM is the largest unified representation in long-term memory that can be used to code the memoranda” (p. 1219). For VWM of Chinese characters, our data suggest that long-term knowledge of visual word forms provides this code and that long-term memory, shaped by a participant’s experience, contributes to working memory.

That experts show better working memory than novices has also been shown in other domains. For example, people show better working memory for upright faces with which we are all familiar (i.e., expert) than for inverted faces (Curby and Gauthier, 2007). Similarly, when working memory for cars was tested, car experts showed better memory than car novices, but this advantage disappeared when the cars were presented upside-down (Curby et al., 2009). VWM for famous faces is also better than for unfamiliar faces, and again, the advantage disappears for inverted faces, i.e., in an unfamiliar viewing condition (Jackson and Raymond, 2008). Working memory is also higher for common objects than for color patches if encoding time is long (1 or 2 s) (Brady et al., 2016). Similarly, participants who were familiar with Pokémons memorized more items than people who were unfamiliar with the specific material (Xie and Zhang, 2017b). These results suggest that familiarity with the to-be-memorized material or pre-experimental knowledge of the to-be-memorized items boosts working memory performance. It follows that increasing experience with a specific material should increase working memory performance (Sorensen and Kyllingsbaek, 2012). However, the mechanisms that cause this advantage are controversial.

For example, the mechanism may be a variant of chunking (Simon, 1974). The features of known items can be chunked and represented as one unit, whereas unknown items are represented in smaller pieces. If one assumes that VWM provides a small number of “slots,” a novel item would fill more slots than a familiar one. If the study array depicts four items, working memory may be capable of representing three of these items if they are familiar. However, the capability may be reduced to one item if the items are novel. If a complex novel character is decomposed into part figures and each sub-figure is individualized, each would fill one “slot.” In this way, one item counts functionally as two or three. A consequence is that the other items in the display cannot be represented and are lost.

Barton et al. (2009) argued against this interpretation. They claimed that working memory always represents “objects” as units and that individual features of any object are not distributed to more than one slot. Hence, even when novel items are seen, working memory should represent about three of them. The authors suggest that poor performance may be a consequence of errors in the comparison process. With complex material such as faces and Chinese characters, changes may often be missed because they are small. Should the change be large (e.g., if a character were changed into a cube), it would be detected, and performance would improve, showing memory for about three items. This is exactly what the authors observed (Awh et al., 2007). The authors therefore distinguished between the number of objects that are represented in working memory and the precision or resolution of these representations. The former was estimated from the detection rate of large changes, and this number correlated with capacity estimations gained from simple material, e.g., color patches (Awh et al., 2007). The same result was observed when comparing working memory for upright and inverted faces (Scolari et al., 2008). The authors took this as evidence that expertise enhances the precision of working memory representation but not capacity in terms of the number of “slots.” The number of items represented should be independent of expertise, but unfamiliar items are represented less precisely than familiar ones (Lorenc et al., 2014).

Even though it is highly meaningful that comparison errors are a function of visual similarity between the study item and test item, some authors have argued against this interpretation of the results. Morey et al. (2012) put forward theoretical arguments. They criticized the computation of the precision parameter and argued that it can only be taken as a measure of the individually experienced distractor similarity, not as a quality of working memory. Other counter-arguments were empirical. The probability of detecting a large change does not only depend on the similarity between the study item and test item but also on the perceptual features of the context in which the study item is presented (ensemble representations) (Brady et al., 2019). This controversy makes it obvious that it is not sufficient to speak of capacity in terms of slots. The similarity between a study item and a test item depends on what is represented in memory, and this is a function of the perceiver’s expertise. Therefore, the perceived similarity, not the physical similarity, is what is relevant to the comparison (Zimmer, 2008; Dall and Sorensen, 2019).

This brings another mechanism to our attention. Expertise influences the quality of perceptual processing. In the field of expertise, stimuli may be encoded by comparisons with templates in long-term memory. With characters, these are word form entries (Dehaene et al., 2005), which are activated via radicals (Perfetti et al., 2005; Chen and Yeh, 2015). Experts can represent a character as a perceptual token of a word form type. This token is constructed by interactive activation of perceptual output and representations of the orthographic word forms (types) in long-term memory (Leck et al., 1995). In the bottom–up stream, orthographic features, e.g., the number of strokes (Sze et al., 2015), influence character encoding, whereas qualities of word forms, e.g., character frequency or diagnostic features of stroke patterns, influence perception in the top–down stream. As a consequence, Chinese literates may need less perceptual support for identification than novices and fill in information that they missed. In contrast, Chinese illiterates cannot make use of word form knowledge. For these people, processing is oriented to the stroke level (Yeh et al., 2003). They can represent items based only on the bottom–up stream, which makes perception more demanding and often causes incomplete representations that cannot be compensated by long-term knowledge. One consequence is that novices show poorer memory for visually complex characters than for simple characters, a factor that is less relevant for Chinese literates (Zimmer and Fu, 2008; Sun et al., 2011).

Collectively, these data suggest that word form knowledge makes an important contribution to character encoding, and we hypothesize that it also adds to the expertise advantage in VWM. Initial support for this claim is the observation that acquiring long-term knowledge of characters enhances VWM for these characters compared to novel characters and changes neural activity in the visual word form area (VWFA) (Zimmer et al., 2012). The series of experiments reported in this paper should provide further evidence for this word form token hypothesis and the argument that the advantage of Chinese literates in VWM for Chinese characters is a consequence of word form knowledge in long-term memory.

A third route by which expertise can influence VWM performance is a difference in low-level visual processing. The visual processing demands of Chinese characters are high, giving orthography a special relevance (Perfetti et al., 2013). Characters often consist of many strokes, and increasing the number of strokes increases a character’s complexity. Perimetric complexity—a geometrically defined measure of a figure’s complexity—is clearly larger for Chinese characters than for standard fonts of Latin alphabets (Ngiam et al., 2019). In a study by Ma and Chuang (2015), characters with many strokes or stroke crossings as well as enclosed characters were judged as subjectively the most complicated. In another study, complexity—defined by the number of strokes—had similar effects on search times in experts and novices (Yu et al., 2018), which suggests that complexity has an early effect in visual processing. If perception of complex characters is a demanding task, it is conceivable that extended practice in identifying such visually dense stimuli provides Chinese literates with enhanced perceptual skills (McBride and Wang, 2015). Some results speak in favor of this possibility. Visual–spatial abilities were found to be positively correlated with the learning of Chinese characters (McBride-Chang et al., 2005). Additionally, learning to read enhances perceptual skills (McBride-Chang et al., 2011). In a cross-cultural study, it was observed that Chinese scholars exclusively outperformed Greek scholars in visual/spatial processing tasks (Demetriou et al., 2005). As yet, however, we cannot provide evidence for this knowledge transfer hypothesis. In our previous studies, Chinese literates did not show an advantage in VWM for other types of complex visual material that were structurally similar to Chinese characters (e.g., Zimmer and Fu, 2008; Sun et al., 2011).

Although evidence for the transfer of encoding skills to novel visual material is lacking, it is still possible that experts process items within their field of expertise differently compared to novices. For example, experts might focus specifically on diagnostic features. In Wagar and Dixon’s (2005) study, for instance, Greeble experts detected changes of diagnostic features more frequently and non-diagnostic features less frequently than novices, who recognized both changes equally often. Another possibility is that experts represent more high spatial frequencies compared to novices. It has been suggested that items are initially represented in working memory at a coarse level and more detailed information is then added (Gao et al., 2009, 2010; Gaspar et al., 2013). According to such models, the aggregation of information over time proceeds from global shape to fine details (Alvarez and Cavanagh, 2008; Xu and Chun, 2009). Because the availability of long-term knowledge influences the speed of this process (Brady et al., 2009), it may also change the level (the resolution) that is finally reached if known visual material is encoded.

Finally, one can think of other, non-visual influences of expertise on working memory. For example, for Chinese literates, words have phonology and meaning. However, even if they are available, phonological codes do not seem to be relevant in VWM tasks. The presence or absence of articulatory suppression was found to have no influence on VWM performance (Morey and Cowan, 2004; Jackson and Raymond, 2008; Sun et al., 2011; Xie and Zhang, 2017c; Ngiam et al., 2019). It is probable that timing and the large number of trials do not favor a naming strategy. However, articulation impairs memory if it has side effects on visual processes, e.g., if words can be imagined (Mate et al., 2012). Also, a verbal preload does not change the working memory effects (Luck and Vogel, 1997; Vogel et al., 2001; Wagar and Dixon, 2005). Therefore, we do not believe that characters are phonologically recoded. The usage of semantic codes will be discussed in Experiment 4.

Obviously, several non-exclusive mechanisms can contribute to the expertise advantage in temporary memory for Chinese characters. In the following experiments, we investigate the relevance of the different components. Experiments 1 to 4 involved change detection tasks with different types of material. Experiment 5 consisted of a training study in which we taught a set of characters and compared the VWM for trained and novel characters.

## General Procedure

All participants were either native Chinese speakers (experts) or Germans with no knowledge of Chinese language (novices). In Experiment 5, participants were native German speakers. The trial structure matched the standard procedure of a change detection task. A study display was presented for a short period. It depicted a variable number (set size) of “objects” (characters, figures, color patches). After a short retention interval, a single “object” was shown, and participants were required to decide whether this item had been presented during the study or something had changed. The experiments were controlled by E-Prime 2. The details and material of the experiments are presented in the Supplementary Material to this paper. The studies were approved by the ethics committee of the Fakultät für Empirische Humanwissenschaft, Saarland University, and all participants gave their informed consent.

We counted the proportions identified correctly and calculated corrected recognition scores (Pr) for each condition. Pr is the difference between the proportion of hits (detected changes) in each change condition and the proportion of false alarms (change responses to unchanged items) to all items in the same stimulus category, e.g., all unchanged characters or color patches. The Pr data were analyzed by repeated measurement analyses of variance (ANOVA). If sphericity was violated, a Huynh–Feldt correction was applied and epsilon reported.

## Experiment 1

The first experiment tested whether novices store fewer items of a study display compared to experts. If a complex item “consumes” full capacity, novices should represent only one item at the expense of other displayed items. For example, Chinese illiterates might represent parts of one item in three “slots” and fully ignore other items. However, it is also possible that novices represent about three items similarly to Chinese literates, but only one might embody details, while the other items are represented merely by coarse information, which is rarely appropriate to detect a character change. Should coarse information be sufficient, the change would be detected in more than one item. In order to show this, it is necessary to measure memory performance for Chinese characters with “big” changes. We presented characters in color ink and then changed the color of an item from study to test. If Chinese illiterates store only one item of the study array, they would miss even the color change in a character because the “object” that had changed color was not represented in working memory at all. In contrast, if the low performance for characters is a matter of “resolution,” a color change (big change) would be detected, but a character change (small change) would not be detected. To test this, we ran a change detection experiment with colored Chinese characters in which the character, its color, or both could change. For controls, color patches were presented in a fourth condition.

### Participants

Twenty-eight Chinese students were tested in Beijing, and 20 German students from Saarland University also took part in the experiment. One Chinese participant was excluded due to below-chance performance.

### Design and Procedure

The study was a 2 × 4 mixed design with expertise (Chinese, German) and change type as the factors. Change type included four levels: three with character arrays as study material (the character changed, character’s color changed, both features changed) and one with color patches (color changed). The set size was four, the study time 500 ms, and the retention interval 1,000 ms.

### Results and Discussion

**FIGURE 1:**
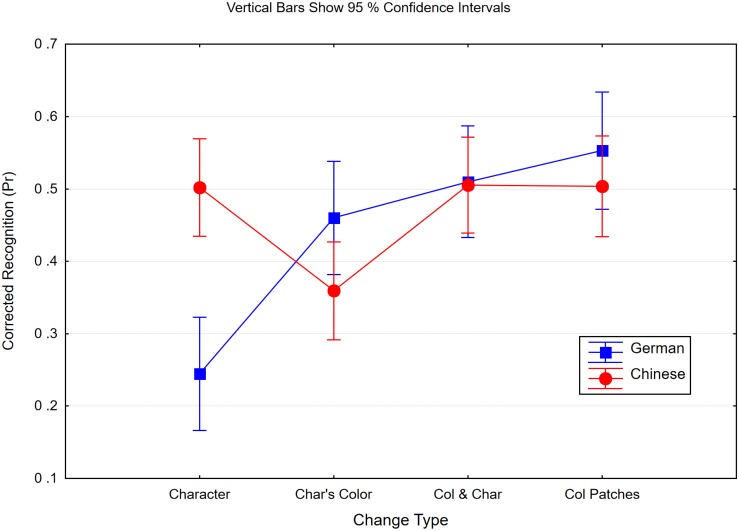
Pr scores of Chinese and German participants in the four different change conditions of Experiment 1. When characters were studied, either the probe character’s identity (character), its color (Char’s Color) was changed, or both features (Col and Char) were changed. In the fourth condition, color patches were studied and one of the colors was changed (Col Patches). Within subject confidence intervals are in both groups ±0.05.

We calculated Pr, as shown in Figure 1. Having calculated the proportion correctly identified in the different change conditions, we subtracted from each accuracy rate the rate of change responses to no-change trials (false alarms) with the same item material, i.e., characters or color patches. In an ANOVA with expertise (Chinese, German participants) and change type as factors, the two factors interacted [*F*(3,135) = 23.10, *p* < 0.0001, *η_*p*_*^2^ = 0.34, mean standard error (*MSE*) = 0.013, ε = 0.88). In both groups, the simple main effects were significant [German: *F*(3,57) = 37.12, *p* < 0.0001, ε = 0.73; Chinese: *F*(3,78) = 9.69, *p* < 0.0001, ε = 0.76]. However, the profiles of performance over change types were different. Germans showed poor memory only when characters changed (small change) but not when their color changed (big change). However, in one of the conditions in which color was changed, the two groups differed. Chinese detected the changed color of an unchanged character worse (Δ = 0.15) than any other change (*p* < 0.0002 in pairwise comparisons). All other conditions did not differ from each other (*F* < 1).

We inferred that novices represent nearly the same number of “objects” if they encode Chinese characters as they do when they encode visually less complex material (color patches). Therefore, the poor character memory of novices was not due to a general failure of encoding the complex items, i.e., “missed objects.” More likely, at a coarse level, all participants encode as many items as they can individualize (Xu and Chun, 2009), but novices represent fine details of one item only or thereabouts, whereas experts do this for nearly all items of the study display. This is not a consequence of the encoding time being too short for the unfamiliar characters. The encoding rate for unfamiliar characters has been estimated at 12 items per second (Ngiam et al., 2019), and in a study with variable and masked presentation time, performance increased with time but reached an asymptote at 450 ms (Sun et al., 2011). A study time of 500 ms should therefore be long enough to encode four items.

## Experiment 2

If the number of represented objects is no different but experts represent “objects” in more detail, this may be due to a general competence or may be restricted to the field of expertise. Their long experience with complex visual input may have enhanced the perceptual skills of Chinese literates when processing fine details in general. In this case, we should see better memory also for other types of visual material if small changes are to be detected.

Addressing a similar question, Fukuda et al. (2010) used a change detection task with big changes (to shape) or small changes (to the inner patterns of shapes). They constructed figures of different shapes (oval, rectangle, and so on) with differing inner patterns (e.g., two crossing versus two parallel lines). In change trials, an altered outline established a big change, whereas a changed inner pattern was a small change. Memory performance in big-change trials estimated “capacity,” while performance in small-change trials estimated “precision.” In Experiment 2, we used the same manipulation. If Chinese literates have generally enhanced perceptual skills for fine details, they should have better memory for the inner patterns of shapes compared to Germans. With big changes, we do not expect any memory differences.

Additionally, we wanted to replicate the results of Experiment 1. For that purpose, we presented characters in different colors as in Experiment 1, but in Experiment 2, they were also presented in different fonts. Each of these features could change. If Chinese literates represent perceptual details of characters, we should see a memory advantage for experts even if a character’s font was changed. If, however, Chinese literates represent the characters as tokens of visual word forms, font memory should be poor, though the characters’ identities should be remembered.

### Participants

Forty-four students from Saarland University took part in the experiment. Twenty-four were native Chinese speakers who studied at Saarland University, and the remainder were native German speakers with no experience of Chinese language.

### Design and Procedure

On the study display, we presented four “objects” (geometrical figures, color patches, or characters) for 500 ms. For the test, one “item” was shown. Figures and color patches defined a 2 × 3 design, with expertise (Chinese, German) and change type (shape, pattern, color patch) as factors. Characters also defined a 2 × 3 design, with expertise (Chinese, German) and change type (color, character, font) as factors. All six trial types were mixed and presented in random order. In change trials, only one feature was altered.

### Results and Discussion

**FIGURE 2:**
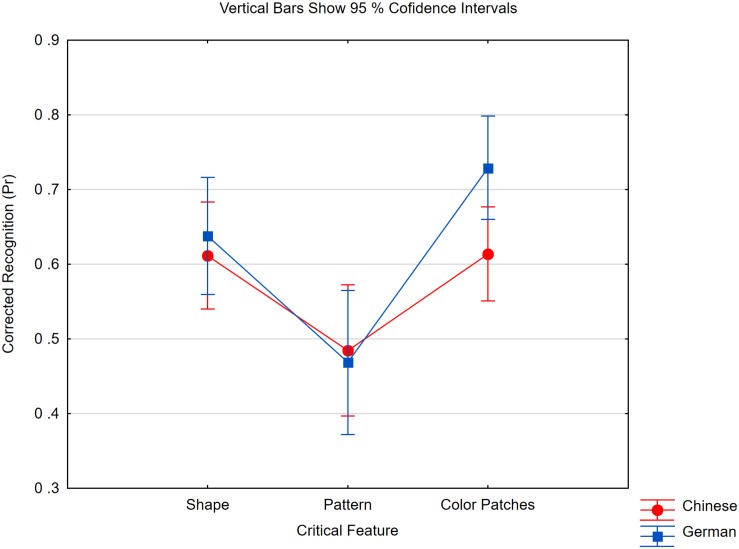
Pr scores of Chinese and German participants in the three change conditions of Experiment 2 if geometric material was studied. Either one figure’s outline (Shape) or its inner pattern (Pattern) was changed or color patches were studied and one of the colors was changed (Color Patches). Within subject confidence interval is ±0.05 in the Chinese group and ±0.06 in the German group.

We analyzed the Pr scores in two 2 × 3 ANOVAs with expertise (Chinese, German) and critical feature (shape, pattern, color patch; or color, character, font) as factors. As shown in Figure 2, with geometrical material, we obtained a clear effect of the changed feature [*F*(2,84) = 31.11, *p* < 0.0001, *η_*p*_*^2^ = 0.43, *MSE* = 0.015] and an interaction with expertise [*F*(2,84) = 3.37, *p* < 0.05, *η_*p*_*^2^ = 0.07, *MSE* = 0.015]. The interaction was due to the fact that Germans showed better working memory for color patches than the Chinese did [*t*(42) = 2.45, *p* < 0.05]. Because a difference in color memory was not observed in other experiments from our lab (Zimmer and Fu, 2008; Sun et al., 2011), we considered this result as accidental. When we excluded the color patch condition, the interaction disappeared (*F* < 1). Both groups detected pattern changes less well than shape changes [*F*(1,42) = 30.62, *p* < 0.0001, *MSE* = 0.016, *η_*p*_*^2^ = 0.42]. Hence, Chinese literates did not show an advantage in detecting small changes to figures.

**FIGURE 3:**
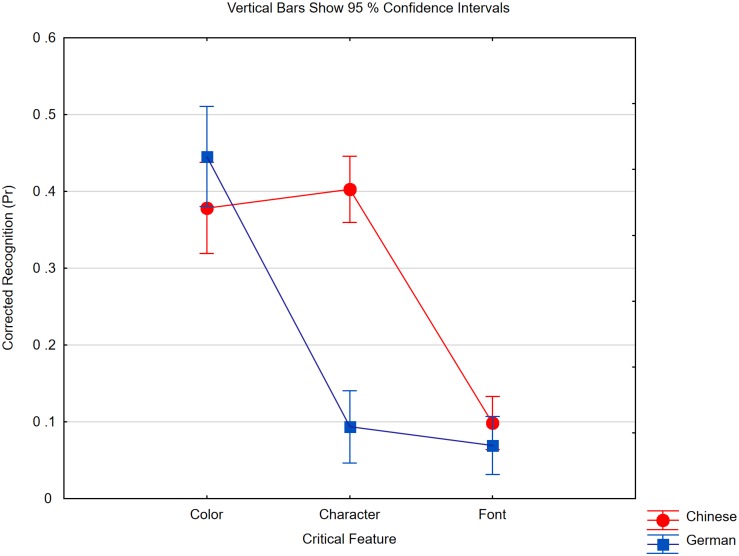
Pr scores of Chinese and German participants in the three change conditions of Experiment 2 if characters were studied. The critical feature denotes the one that was changed and it was either the probe character’s color (Color), identity (Character) or font (Font). Within subject confidence interval is ±0.05 in the Chinese group and ±0.06 in the German group.

As shown in Figure 3, in the character conditions, we obtained a very strong effect of the changed feature [*F*(2,84) = 112.75, *p* < 0.0001, *η_*p*_*^2^ = 0.73, *MSE* = 0.010] and an interaction with expertise [*F*(2,84) = 40.04, *p* < 0.0001, *η_*p*_*^2^ = 0.49, *MSE* = 0.010]. The interaction was driven by two influences. First, the two groups did not differ if a change to a character’s color had to be detected [*t*(*42*) = 1.53, *p* = 0.13], but Chinese participants detected a character change much better than German participants [*t*(*42*) = 9.79, *p* < 0.0001]. This replicates the results of Experiment 1. However, both groups did not differ in being very poor at detecting changes to a character’s font [*t*(*42*) = 1.15, *p* = 0.25], even though Chinese participants (but not Germans) remembered the characters’ identities.

Obviously, literates in Chinese represented abstract word forms but no detailed visual information on the presented characters. One might argue that this was only strategic: they habitually focused on a character’s shape and ignored its font. In that case, however, it would be surprising that characters’ colors were remembered. Nevertheless, to exclude the possibility that we unintentionally missed the experts’ memory advantage for fonts, we tested memory for fonts again in Experiment 3, but this time, processing was explicitly focused on the relevant feature.

## Experiment 3

### Participants

Forty students from Saarland University took part in the experiment. Half were native Chinese speakers studying in Germany, and half were native German speakers.

### Design and Procedure

On the study display, three characters were visible for 500 ms (for details, see the Supplementary Material). The participants’ tasks changed block-wise every 36 trials. In a block, they had to spot a change to a character or a font, or they had to spot both features. We therefore had four types of stimulus matches between study and test: (a) no change; (b) the character was changed but the font was the same; (c) vice versa; and (d) both features were changed. Because each feature changed half of the time irrespective of the task, we could also test irrelevant change effects. If participants could focus successfully on the relevant feature, the match of the irrelevant feature should not matter.

### Results and Discussion

**FIGURE 4:**
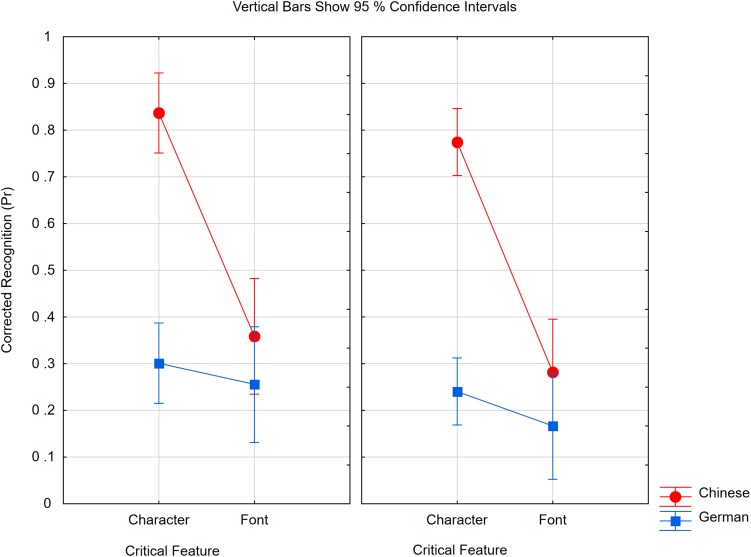
Pr scores of Chinese and German participants in Experiment 3 if only one feature was task relevant (critical feature) which was either the character or the font. In the left panel the irrelevant feature was the same, in the right panel the irrelevant feature was changed. The within subject confidence interval is ±0.08 for the German participants and ±0.07 for Chinese participants.

Corrected recognition scores were calculated by subtracting the proportion of false alarms (change decisions to no-change items) from the proportion of detected changes at the same level of irrelevant feature and task. We first analyzed the trials in which only one feature was relevant. An ANOVA was performed with expertise (Chinese, German), change type (character, font), and status of irrelevant feature (same, changed) as the factors. The results of Experiment 2 were fully replicated, as shown in Figure 4. We obtained a highly significant interaction between expertise and change type [*F*(1,38) = 46.17, *p* < 0.0001, *η_*p*_*^2^ = 0.55, *MSE* = 0.039]. Chinese literates detected character changes (0.81, SE = 0.04) much better than Chinese illiterates (0.27, SE = 0.06). In contrast, the experts’ memory for fonts (0.32, SE = 0.05) was not significantly better than the novices’ memory (0.21, SE = 0.05) [*F*(1,38) = 2.19, *p* = 0.15, *η_*p*_*^2^ = 0.05, *MSE* = 0.030]. The status of the irrelevant feature did not interact with any other factor (*F* < 1), but decisions were more accurate for unchanged (0.44, SE = 0.03) than for changed (0.37) irrelevant features [*F*(1,38) = 6.37, *p* < 0.05, *η_*p*_*^2^ = 0.14, *MSE* = 0.033].

**FIGURE 5:**
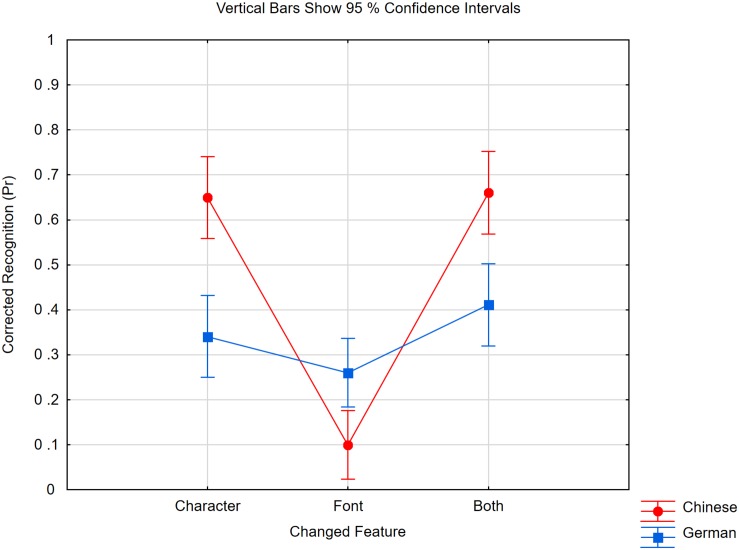
Pr scores of Chinese and German participants in Experiment 3. Participants spotted both features (character and font) and the probe character changed either its identity (Character), font (Font), or both features (Both). The within subject confidence interval is ±0.06 for the German participants and ±0.07 for Chinese participants.

We then analyzed the condition in which both features had to be attended. Figure 5 depicts the respective data as a function of change type. In an analysis with expertise (Chinese, German) and change type (character, font, both) as factors, we obtained a strong interaction [*F*(2,76) = 32.81, *p* < 0.0001, *η_*p*_*^2^ = 0.46, *MSE* = 0.020]. The better working memory performance of Chinese literates was confined to conditions in which the character changed [*F*(1,38) = 23.77, *p* < 0.0001]. If only the font was changed, Chinese participants were even worse than the Germans [*t*(38) = 3.01, *p* < 0.005]. However, within the German group, the change type had an effect [*F*(2,38) = 6.66, *p* < 0.005, ε = 0.997]. A Bonferroni test revealed that memory was better if both features had changed (0.41, SE = 0.05) than if only one had changed, but memory for characters (0.34, *SE* = 0.03) and fonts (0.26, *SE* = 0.04) did not differ.

Even though we highlighted the relevance of fonts and the test conditions were blocked, we did not find evidence for enhanced memory for fonts among the experts. In both feature blocks, Chinese showed even worse memory performance if only a character’s font had changed. This is plausible if we assume that both groups have poor memory for fonts but that, in contrast to Germans, Chinese people recognized the character as the same. A consequence of this would be the erroneous no-change decisions. We concluded from these results that expertise did not enhance the representation of low-level details: what was easily detected by Chinese literates (the experts) was the mismatch of the word form.

## Experiment 4

The results of Experiment 4 should provide arguments against an alternative interpretation of the data. Experts have additional codes available that do not exist for novices and that have the potential to support memory. They can name the “objects” and use a phonological code for storage. The observation that articulatory suppression does not change the results (e.g., Curby and Gauthier, 2007; Sun et al., 2011; Sense et al., 2017) is an argument against a naming strategy. However, for Chinese literates, characters also have meaning, and meaning may be available even under articulatory suppression (Bourassa and Besner, 1994; Shivde and Anderson, 2011; Baier and Ansorge, 2019). The results of Experiment 4 provide evidence that Chinese literates still use VWM and not the meaning of the characters.

To test this hypothesis, we manipulated the visual and semantic similarities between the studied and the changed character. Memory performance should be poor if the similarity between the item held in memory and the presented foil is high. If the participant uses a visual memory code, visually similar foils should be erroneously considered as old (Chen et al., 1995; Leck et al., 1995). If the code is semantic, semantically similar items should cause poor performance because the distractor has the same meaning as the study item. As a test, therefore, we compared change detection performance with characters under high and low visual or semantic distractor similarity.

Additionally, we presented pseudo characters as study material. Pseudo words combined two radicals of different real characters. These “artificial characters” do not exist in Chinese language and therefore have no meaning. This allowed us to manipulate the visual similarity in the change condition in the absence of meaning. We did so by replacing one of the radicals of a studied pseudo word with a visually similar or dissimilar radical but still generating pseudo words. If memory in the visually similar condition is impaired to a similar extent for characters and pseudo characters, this would suggest that working memory for characters is based mainly on visual information.

### Participants

Twenty students from universities in Beijing took part in this experiment.

### Design and Procedure

**FIGURE 6:**
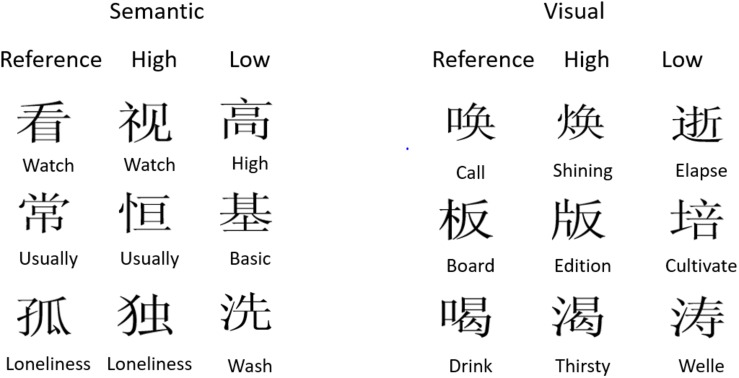
Examples of the study-test variation in Experiment 4. Three examples (rows) of each item type are shown together with its foils that were presented in a change trial. The three columns show the studied item (reference), and foils with a high or low similarity (on the left for the semantic character set and on the right for the visual character set). The English translations were not shown in the experiment. The full material is presented in the Supplementary.

Two sets of 10 characters were selected, defined by the type of similarity (as shown in the Supplementary Material). In the visual set, for each study item, we selected a foil that had a different meaning but a shared radical; in the semantic set, the changed item was a synonym of the study item without shared visual features (see Figure 6 for examples). Also, highly similar foils to pseudo words shared a radical with the study item, whereas the dissimilar items did not share features with the study item. In order to minimize strategic influences, presentation time was only 200 ms, and an articulatory suppression task was applied (see the Supplementary Material).

### Results and Discussion

**FIGURE 7:**
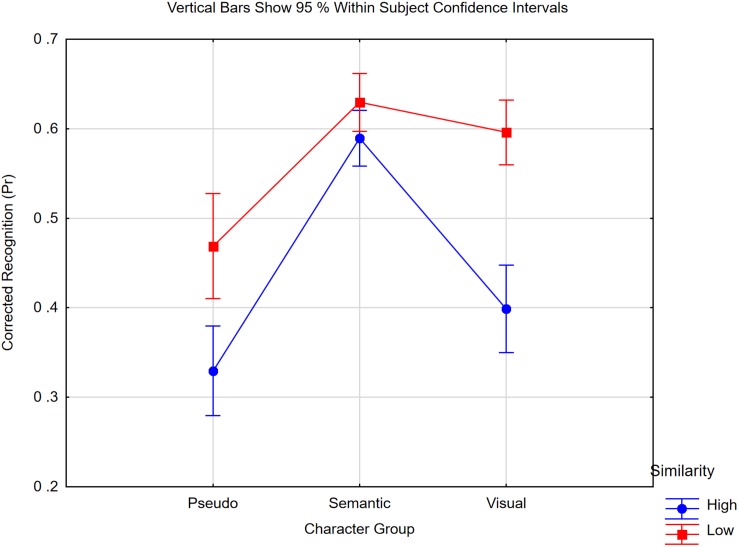
Pr scores of Chinese native speakers for detecting changes in the three different character groups (Pseudo, Semantic, Visual) and the two distractor similarity levels (high, low). “Low” means no feature overlap, “high” means overlap of visual features in the pseudo and visual group, and shared semantic meaning in the semantic group. Confidence intervals are based on within subject variance following Morey (2008).

We calculated for each change type corrected recognition scores, as shown in Figure 7. A 3 × 2 repeated measurement ANOVA was conducted with character set (pseudo, semantic, visual) and similarity level (high, low) as the factors. The results showed a strong interaction [*F*(2,38) = 11.36, *p* < 0.0005, *η_*p*_*^2^ = 0.37, *MSE* = 0.005]. Visual similarity (pseudo: 0.17; characters: 0.20) impaired memory much more than semantic similarity [0.04; *t*(19) = 4.35, *p* < 0.0005], though the latter effect was nearly significant [*t*(20) = 2.06, *p* < 0.06].

In a follow-up, we analyzed the character conditions in a 2 × 2 ANOVA with type of similarity (semantic, visual) and similarity level (high, low) as the factors. Again, the interaction was significant [*F*(1,19) = 19.13, *p* < 0.0005, *η_*p*_*^2^ = 0.50, *MSE* = 0.007]. Visually similar foils showed the worst memory compared to all other conditions (*p* < 0.0001). When we compared only the visual conditions in a 2 × 2 analysis with word form (pseudo, characters) and similarity level (high, low) as factors, the main effects of word form [*F*(1,19) = 8.13, *p* < 0.0001, *η_*p*_*^2^ = 0.30, *MSE* = 0.024] and visual similarity [*F*(1,19) = 49.35, *p* < 0.0001, *η_*p*_*^2^ = 0.72, *MSE* = 0.012] were both significant, but the interaction was not significant [*F*(1,19) = 3.11, *p* = 0.10, *η_*p*_*^2^ = 0.15].

Pseudo characters were memorized worse than real characters, and both were strongly and to the same extent influenced by the visual similarity of the foils. Semantic similarity had only a weak effect. This speaks in favor of the assumption that even Chinese literates represent the items in a change detection task as visual code in working memory.

## Experiment 5

Collectively, the above experiments provide evidence for the word form token hypothesis, which assumes that characters are represented in VWM as tokens of word forms stored in long-term memory. In this experiment, we investigated neuropsychological evidence for this claim by demonstrating the positive effect of making available this knowledge of word forms.

Even though practicing items sometimes have no effect on VWM (Olson and Jiang, 2004; Eng et al., 2005), research has found that performance in a trained working memory task usually increases with practice (Redick et al., 2015). This was also found in VWM (Gaspar et al., 2013; Kundu et al., 2013; Owens et al., 2013; Schwarb et al., 2015) and also with Chinese characters (Zimmer et al., 2012). However, it has not yet been shown that providing word form knowledge outside of a working memory task also enhances performance. We wanted to demonstrate this in a brain imaging experiment.

The neural consequences of practice can be various (Kelly and Garavan, 2005). In a task with low demands, Heinzel and colleagues observed that after training, participants exhibited less neural activity than before training to achieve the same level of performance. The authors explained this effect by higher neural efficiency (Heinzel et al., 2014, 2016). The same result was observed in a previous study using Chinese characters (Zimmer et al., 2012). A similar effect was also expected in this experiment, specifically in two regions: the intraparietal sulcus (IPS) and the fusiform cortex (FFC).

The IPS is a core structure of working memory (Xu, 2007; Xu and Chun, 2009; Xu and Jeong, 2016). It contributes to individuation and identification of stimuli in working memory tasks and is involved in focusing attention on memorized stimuli. Activity in the IPS was found to increase with set size and level off at working memory capacity (Xu and Chun, 2006; Robitaille et al., 2010). Hence, with small set sizes, we expected less neural activity for trained than for untrained characters. With larger set sizes, neural activity for both sets of items should approach a common maximum.

Additionally, according to the sensory recruitment hypothesis (Serences, 2016), those structures that encode the stimuli during perception should represent the items in working memory. For characters, this should be the VWFA in the FFC, as was shown for Western languages (Dehaene et al., 2002; Liu et al., 2008; Dehaene and Cohen, 2011) and for Chinese (Xue et al., 2006; Xue and Poldrack, 2007). If trained characters are recognized with less effort than untrained ones, we should see a reduction in neural activity within the VWFA.

In order to test this hypothesis, German participants (novices) learned the orthography of 12 Chinese characters without any information on pronunciation or meaning. We then compared memory for trained and untrained characters in a change detection task.

### Participants

Twenty-four students from Saarland University took part in the experiment. Two were excluded from the analysis because of below-chance performances. None of the participants had pre-experimental experience with Chinese language, and no participant took additional time to learn the word forms or their meanings outside of the practice sessions.

### Design and Procedure

Participants were presented repeatedly with a set of 12 characters in 12 training sessions (3 per week), in which participants saw animated writing videos and actively copied the characters. In developing the training materials, particular attention was paid to the guidelines that writing videos should establish “a high quality representation of the visual–spatial structure of the character and its orthography” (Cao et al., 2013a, b, p. 1670), especially when combined with animation (Xu et al., 2013). In each writing animation of the training material, one character was drawn stroke by stroke. The training sessions consisted of stroke copying, character drawing, a one-back task, and, from session 7, additionally, a written free recall. Details can be found in the Supplementary Material.

Working memory performance was tested in a change detection task with a set size of one to three in a 3T scanner. In the pre-test before training, all items were novel; in the post-test, half were trained and half untrained. The items that were trained and tested as novel material were counterbalanced across subjetcs. Presentation time was 1,000 ms. After a retention interval of 4,000 ms, one central test stimulus was presented. The displayed “items” were characters or random patches, i.e., squares of the same size as the characters, filled with spatially randomized pixels of the characters. In the MR analyses, the parameters per set size were estimated as contrasts between the character condition and the random patch condition to remove non-specific visual activity.

### Results

#### Behavioral Measures

To test knowledge acquisition of the trained word forms, we analyzed participants’ performance in the free recall task. In the first recall (session 7), participants could already write down from memory 10.6 of the 12 characters on average. By session 12, their performance had increased to 11.6 out of 12 [*t*(21) = 3.31, *p* < 0.005[.

**FIGURE 8:**
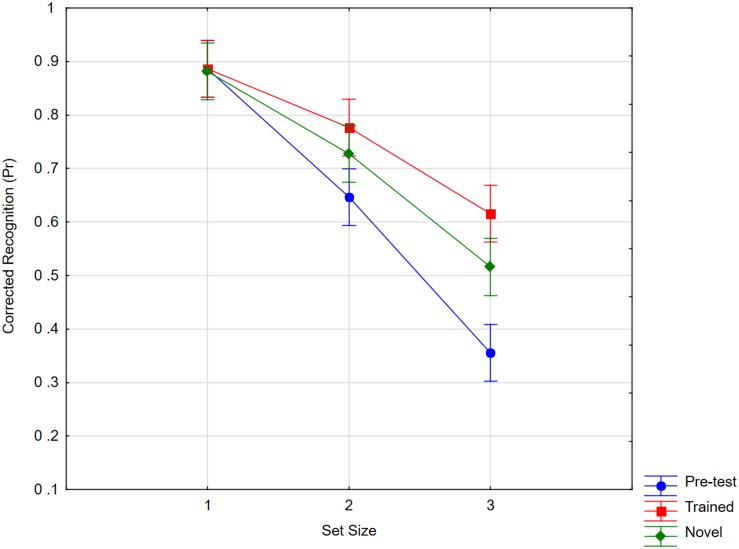
Recognition scores as a function of set size (1, 2, 3) and item status (pre-test, trained, novel). Whiskers show within subject confidence intervals based on the error term of the interaction according to Loftus and Masson (1994).

As shown in Figure 8, Pr scores in the change detection task were analyzed in a 3 × 3 analysis with set size (1, 2, 3) and status (pre-test, novel, trained) as the factors. The results showed a significant interaction [*F*(4,84) = 6.70, *p* < 0.001, *η_*p*_*^2^ = 0.24, *MSE* = 0.014, ε = 0.85]. Such an effect was also seen when we compared pre-test memory for novel characters with post-test memory [*F*(2,42) = 4.33, *p* < 0.05, *η_*p*_*^2^ = 0.17, *MSE* = 0.017, ε = 0.85]. This is an unexpected general effect of practice. Additionally, the results showed an item-specific training effect. Memory performance at set size one showed ceiling effecs already in the pre-test, and we therefore only contrasted trained with novel characters at set sizes of two and three, trained characters were memorized better than novel ones [*t*(21) = 1.90, *p* < 0.05, single sided]. The estimated “capacity” in terms of the number of items that could be stored in working memory was 1.1 at pre-test and, at post-test, 1.5 for novel characters and 1.8 for trained characters.

#### Brain Imaging Data

Bold responses were analyzed in SPM 12. Pre-processing followed the standard procedure. In the design matrix of the model, only trials with correct responses were included. We were interested in set size and training effects during the retention interval within the IPS and FFC. For this analysis, we modeled the activity during the 4 s retention interval as a boxcar function using the canonical double-gamma hemodynamic response function (HRF) functions of SPM. Due to the predicted changes in specific brain areas, we estimated parameters for average activity within pre-specified regions of interest (ROIs) using the MarsBAR ROI toolbox. We extracted for each participant the parameter estimates for the six combinations of set size and item status within these ROIs and used these scores as dependent variables in the ANOVAs. For time course analysis within these ROIs, we extracted parameter estimates for nine repetition time (TR) points beginning with the retention interval. In the time course analysis, we modeled activity as finite impulse responses; this method does not constrain the shape of the time course.

#### Task-Related Activity During Retention

Xu and Chun (2006) have highlighted the relevance of the inferior IPS (+26/−25, −65/−70, 34/29)^1^ and superior IPS (+26/−21, −52/−66, 45/46) for individuation and identification, respectively. Sheremata et al. (2010) subdivided the IPS into smaller regions, IPS0 to IPS4 and anterior IPS. With regard to visual short-term memory, they argued that set size drives activity in IPS0 (±26, −78, 29), IPS1 (±22, −71, 41), and IPS2 (±18, −63, 52). We therefore defined spheres with a diameter of 5 mm around the coordinates of IPS0 to IPS3 and extracted parameter estimates for average activity within these volumes for the different combinations of set size and character status. We expected to see an increase in activity with set size but less activity for trained than for novel characters, while both would approach a common maximum at high set sizes. We only report activity in the left IPS: the right IPS showed qualitatively the same effects.

**FIGURE 9:**
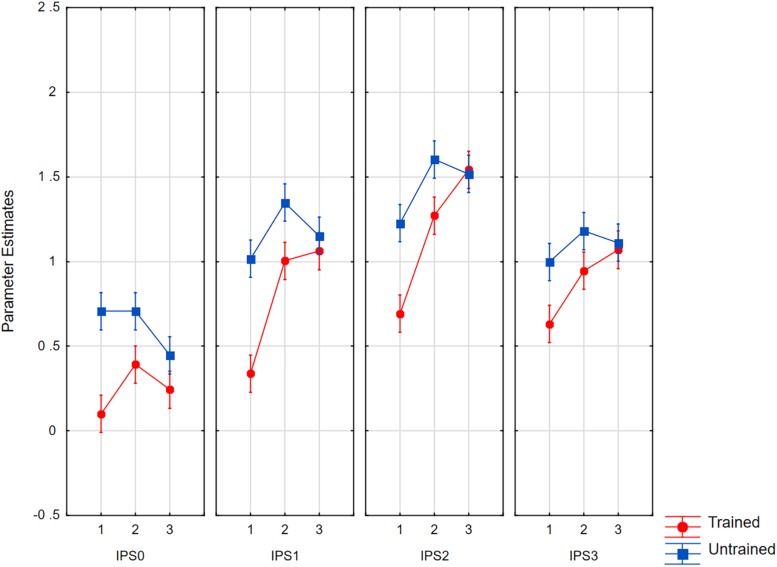
Parameter estimates for average activity in the left IPS0 to IPS3 volumes as a function of set size and item status. Whiskers show within subject confidence intervals based on the error term of the triple interaction according to Loftus and Masson (1994).

Averages per condition (Figure 9) were analyzed in a 4 × 2 × 3 ANOVA with the factors as follows: IPS region (0 to 3); training status (trained, novel); and set size (1, 2, 3). The results showed a significant effect of region [*F*(3,63) = 7.13, *p* < 0.0005, *η_*p*_*^2^ = 0.25, *MSE* = 2.45, ε = 0.81], which interacted with set size [*F*(6,126) = 3.57, *p* < 0.007, *η_*p*_*^2^ = 0.15, *MSE* = 0.247, ε = 0.74]. The interaction was due to the fact that the strongest set size effects were seen in IPS1 and IPS2, which roughly correspond to the inferior and superior IPS discussed by Xu and Chun (2006). With set sizes of one and two, activity was lower for trained than for novel material [*F*(1,21) = 9.41, *p* < 0.006]. For trained characters, the activity per set size followed the order 1 < 2 < 3, whereas for untrained material, the order was 1 < 2 = 3. In IPS0, only the training effect was significant [*F*(1,21) = 7.71, *p* < 0.05; set size: *F* < 1].

**FIGURE 10:**
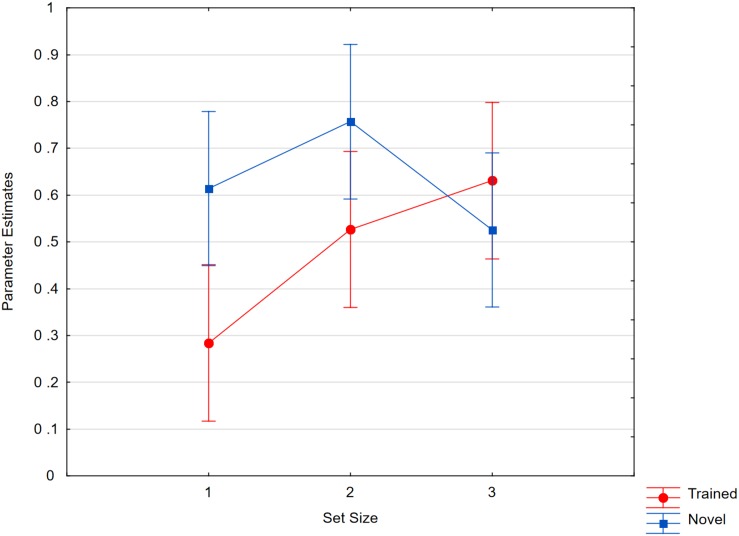
Parameter estimates for activity in the sphere around the center of Cohen’s VWF area as a function of set size and item status. Whiskers are within subject confidence intervals based on the error term of the interaction according to Loftus and Masson (1994).

The average activity in the FFC was estimated within a 5 mm sphere around the coordinates of the VWFA (−42, −57, −15) as defined by Cohen et al. (2002). As shown in Figure 10, we analyzed the extracted values in a 2 × 3 analysis with item status (trained, novel) and set size (1, 2, 3) as the factors. The only significant effect was found in the interaction between the two factors [*F*(2,42) = 4.33, *p* < 0.05, *η_*p*_*^2^ = 0.17, *MSE* = 0.132, ε = 0.68]. For set sizes of one and two (but not set size of three), trained characters showed less activity than novel ones [*t*(21) = 3.09, *p* < 0.005].

#### Time Course of Activity

**FIGURE 11:**
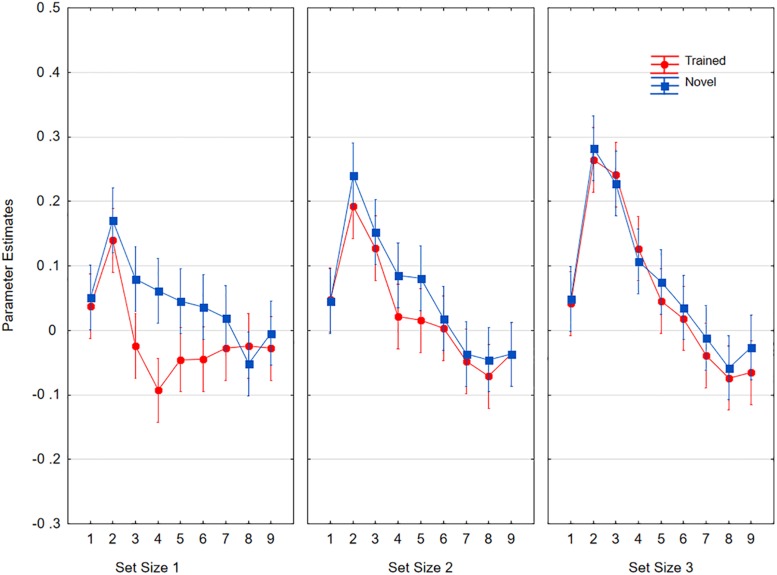
Time course of activity averaged across IPS1 and IPS 2 volumes as a function of item status (trained, novel) and time points (TR1 to TR9) for the three set sizes (1, 2, 3). Whiskers show within subject confidence intervals based on the error term of the triple interaction according to Loftus and Masson (1994).

We then looked at the time course of neural activity within IPS1 and IPS2, the two IPS regions with the strongest effects. We analyzed the data in a 2 × 2 × 3 × 9 ANOVA with the four factors as follows: region (IPS1, IPS2); item status (trained, novel); set size (1–3); and time point (T1 to T9). Because IPS1 and IPS2 showed comparable effects and no interactions, we present in Figure 11 the mean parameter estimates for the average of both IPS regions in steps of TR (1,800 ms), time-locked to the onset of the retention interval. Trained characters elicited less activity in the IPS than novel characters [*F*(1,21) = 6.41, *p* < 0.05, *η_*p*_*^2^ = 0.23, *MSE* = 0.10]. Activity increased with set size (*F*(2,42) = 7.71, *p* < 0.005, *η_*p*_*^2^ = 0.27, *MSE* = 0.070, ε = 0.94] and decreased over time [*F*(8,168) = 28.23, *p* < 0.001, *η_*p*_*^2^ = 0.57, *MSE* = 0.069, ε = 0.55]. Set size interacted with time point [*F*(16,336) = 9.77, *p* < 0.001, *η_*p*_*^2^ = 0.32, *MSE* = 0.017, ε = 0.57], and the three-way interaction was also significant [*F*(16,336) = 2.03, *p* < 0.05, *η_*p*_*^2^ = 0.09, *MSE* = 0.003, ε = 0.50].

In Figure 11, neural activities at each time point are plotted separately for the three set sizes. With small set sizes, starting at the same maximum, the activity returned to baseline more quickly for trained characters than for novel characters. For a set size of three, no differences were observed.

**FIGURE 12:**
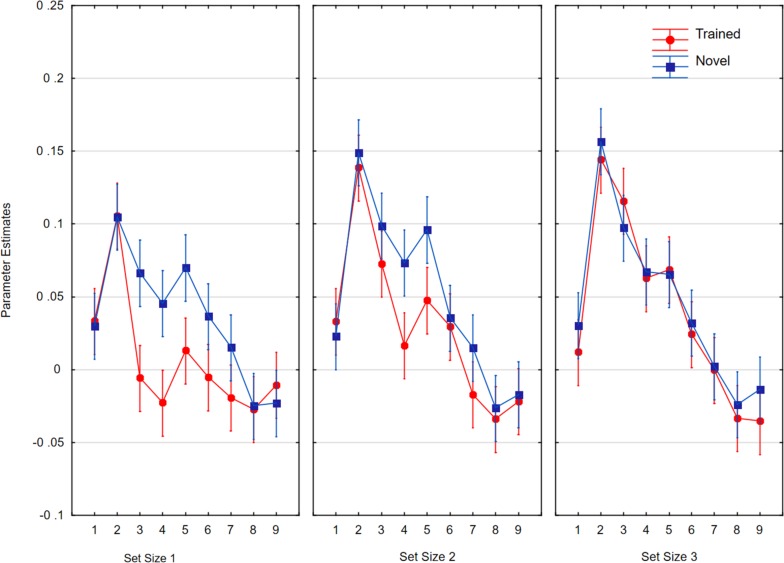
Parameter estimates for the fusiform cluster as a function of item status (trained, novel) and time point (TR1 to TR 9) for the three set sizes. Whiskers show within subjects confidence intervals based on the error term of the interaction according to Loftus and Masson (1994).

For the analysis of time courses in the FFC, we defined the region of interest around the coordinates (−40, −56, −3), which showed the maximum of activity in our participants. As shown in Figure 12, the data were analyzed in a 2 × 3 × 9 ANOVA with item status (trained, novel), set size (1, 2, 3), and time point (1–9) as the factors. Trained items were associated with less activity than novel items [*F*(1,21) = 12.20, *p* < 0.005, *η_*p*_*^2^ = 0.37, *MSE* = 0.008)]. Activity increased with set size [*F*(2,42) = 3.72, *p* < 0.05, *η_*p*_*^2^ = 0.15, *MSE* = 0.015, ε = 0.90] and decreased over time points [*F*(8,168) = 37.68, *p* < 0.001, *η_*p*_*^2^ = 0.64, *MSE* = 0.009, ε = 0.44]. Additionally, there were significant interactions between item status and time point [*F*(8,168) = 2.41, *p* < 0.05, *η_*p*_*^2^ = 0.10, *MSE* = 0.002, ε = 0.55] and between set size and time point [*F*(16,336) = 4.98, *p* < 0.001, *η_*p*_*^2^ = 0.19, *MSE* = 0.002, ε = 0.51]. The triple interactions were also significant [*F*(16,336) = 2.47, *p* < 0.005, *η_*p*_*^2^ = 0.11, *MSE* = 0.002, ε = 0.64]. With regard to the FFC, at set sizes one and two, activity in trials with novel characters stayed high for longer than activity in trials with trained characters.

**FIGURE 13:**
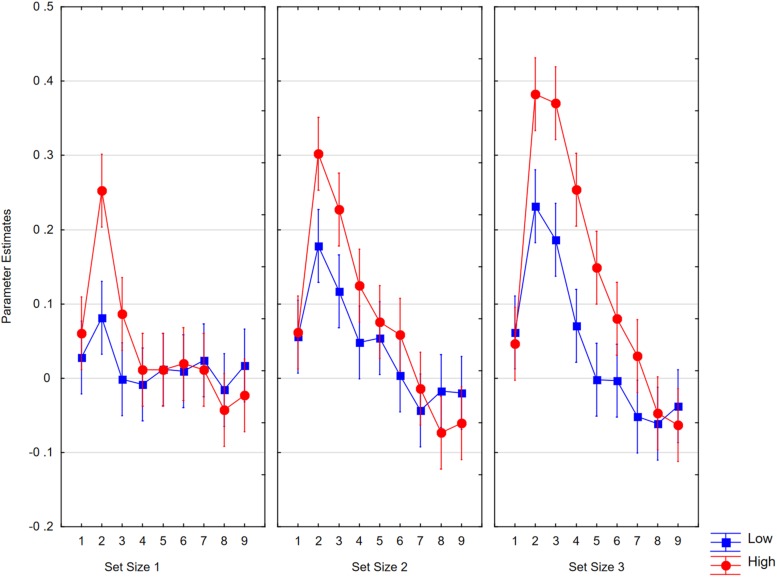
Parameter estimates for activity in the IPS2 volume as a function of performance level (low, high) and time point (TR1 to TR9) for the three set sizes. Whiskers show within subject confidence intervals based on the error term of the interaction according to Loftus and Masson (1994).

#### Performance-Related Neural Activity

If neural activity is relevant to task performance, we should find a relationship with behavioral performance. Because the number of participants was too low for a correlational analysis, we compared neural activity of high and low performers separated at the median performance level in the trained character condition. High and low performers did not differ in performance at pre-test (0.61; 0.65), but at post-test, they differed for trained characters (0.87; 0.65) and for novel characters (0.79; 0.63). We then conducted our time course analyses with the additional factor of performance (high, low). We only analyzed the effects in IPS2 and the FFC, as they were the ROIs showing the strongest effects, and we do not report effects that were not moderated by the performance level factor.

**FIGURE 14:**
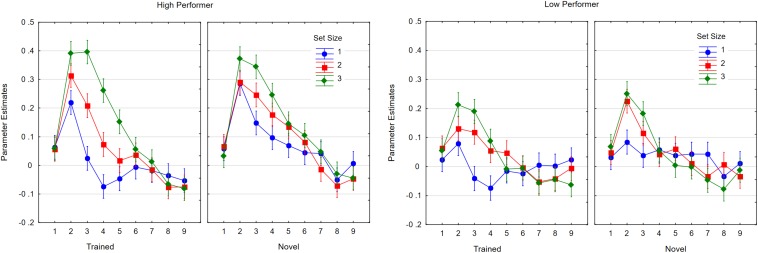
Parameter estimates for activity in the IPS2 volume as a function of time point (TR1 to TR9) and set size (1, 2, 3) when high performers **(left)** or low performers **(right)** memorized trained or novel characters. Whiskers show within subject confidence intervals based on the error term of the highest interaction according to Loftus and Masson (1994).

In the IPS, high-performing participants showed higher neutral activity than low-performing participants [*F*(1,20) = 4.39, *p* < 0.05, *η_*p*_*^2^ = 0.18, *MSE* = 0.0175]. This effect interacted with time point [*F*(8,160) = 3.32, *p* < 0.005, *η_*p*_*^2^ = 0.14, *MSE* = 0.040, ε = 0.52]. The interaction with set size approached significance [*F*(2,40) = 2.88, *p* < 0.06, *η_*p*_*^2^ = 0.13, *MSE* = 0.0396]. The interaction is shown in Figure 13. Although we observed no higher-order interaction, Figure 14 shows the highest resolution of data to illustrate the consistency of results across conditions.

In the fusiform ROI, the only interaction was found between time point and performance level [*F*(8,160) = 5.35, *p* < 0.001, *η_*p*_*^2^ = 0.21, *MSE* = 0.039, ε = 0.59], which was due to enhanced neural activity among high performers exclusively at time point T2 [*F*(1,20) = 19.97, *p* < 0.001]. No other effect with performance level approached significance.

### Discussion

The behavioral results of Experiment 5 showed a training effect only among the high performers. This was not an item-specific effect, because the performance difference was observed for trained as well as for novel characters. However, item-specific neural training effects were observed for all participants.

The IPS showed set size and training effects; additionally, high performers showed a noticeably stronger activity than low performers. We consider the activity in the IPS as being modulated by a participant’s task engagement. The data suggest that, independent of the items’ training status, high performers put more effort into the task than low performers, which probably caused their general memory advantage. We can only speculate why low performers showed a neural but no behavioral training effect. It may be that they paid less attention to a study display if they recognized that it consisted of apparently easier trained items. Such a counterproductive adaptive behavior would use up training gains.

In the IPS and FFC, we saw similar time courses. We interpret the duration of elevated activity as a correlate of effort invested into character encoding and maintenance. In the beginning, in a stimulus-driven manner, set size had the main influence because word form information that provides the training effect only becomes available in the course of encoding. The total amount of effort that is invested into a study display should increase with set size up to the capacity limit. Familiar items needed less effort, which was observed as long as capacity was not exceeded. Although this is speculative, we interpret the fast decay of activity seen for trained characters as an indicator of a shortened or reduced encoding process and/or maintenance process. However, these are *post hoc* interpretations of the time courses, which also depend on the model used for analysis and experimental parameters. In further studies, we plan to vary the length of encoding time and/or of the retention interval in order to gain insight into the origins of time course changes. Additionally, to obtain an estimate of the cognitive effort invested, we should add a independent measure of cognitive effort, for instance, pupil diameters (Kursawe and Zimmer, 2015; Unsworth and Robison, 2016).

## General Discussion

The results of Experiments 1 to 3 demonstrate that Chinese literates had better working memory for Chinese characters than novices without word form knowledge. Experts could hold in memory 2.4 characters on average, whereas novices had a capacity of only one character. Similar results have been reported in other experiments (Sun et al., 2011; Ngiam et al., 2019). The strong effect of visual similarity together with the weak effect of semantic distractor similarity also suggests that experts hold characters as visual representations in VWM (Experiment 4). There was no difference between experts and novices in detecting changes to figures, whether the change was big or small (Experiments 1 and 2). In remembering specific details of characters, experts detected a character change much better than novices, but both groups did not differ in being poor at detecting changes to a character’s font (Experiments 2 and 3) and in being poor at memorizing pseudo characters (Experiment 4). In sum, Chinese literates exhibited a better working memory performance for abstract Chinese word forms, but we did not find indicators of an enhanced working memory in terms of the number of remembered objects and visual features or visual details.

We assume that Chinese literates represent characters as tokens in working memory, each making reference to a word form and additionally representing some coarse episodic information, e.g., color (Gao et al., 2013). Word forms make generic orthographic details available as templates (Dehaene et al., 2005) and allow literates to identify characters quickly. Consequentially, for familiar alphabets, it is not necessary to encode all perceptual details. The important variable for character encoding in working memory is therefore participants’ familiarity with an alphabet (Ngiam et al., 2019). The memory demand of a task is a function of the characters’ perceived complexity, i.e., familiarity (Dall and Sorensen, 2019). Furthermore, a pattern that is partially forgotten can be completed from long-term memory (Thalmann et al., 2019). Comparison errors at the test stage would be unlikely if word forms assign the remembered and perceived character to different categories (words). A possibility to test this would be confidence ratings (Xie and Zhang, 2017a), which could provide evidence that experts’ memory is associated with recollection, whereas novices rely on familiarity. Because word forms do not represent low-level information, experts have no advantage for font memory; for a similar argument regarding visual search, see Baier and Ansorge (2019). Also, pseudo characters with no long-term representation have no expertise advantage. It seems that experts can reliably memorize only one complex unknown character, which is no different from novices. Olsson and Poom (2005) make a similar suggestion concerning another type of material with no categories in long-term memory.

Novices do not have word form knowledge available, and this might render the available encoding time a moderator for the size of the expertise advantage in working memory. This can induce a disadvantage exclusively for novices if they have insufficient time to represent the items in working memory. In a series of experiments using familiar and unfamiliar Pokémons as stimuli, Xie and colleagues reported results that favor this possibility. They observed higher memory capacity for familiar than for unfamiliar figures if the encoding time was constrained (Xie and Zhang, 2017b). At 117 ms encoding time, both item types registered equally poor memory, but with a study time of 314 or 500 ms, familiar Pokémons were remembered better than unfamiliar ones. When the time was increased further to 1,000 ms, the difference disappeared (Xie and Zhang, 2017c, 2018). This suggests that the encoding time provided is the limiting factor. However, other studies could not replicate this. Ngiam et al. (2019) investigated memory for different alphabets. From 120 to 270 ms, performance for all fonts increased. Familiar fonts (Courier, Helvetica, Bookman) had an encoding rate of about 42 items per second and a capacity of about 4 items. Unfamiliar fonts (the handwriting style of the font Künstler; Braille; Hebrew; and Chinese characters) had lower encoding rates (7–15) and capacities (1–1.8). However, the effects of familiarity remained constant between 270 and 600 ms. In a series of experiments, Sun et al. (2011) varied encoding time between 217 and 683 ms. Performance increased up to 450 ms, and the positive effect of familiarity was independent of encoding time. In the experiments reported in this paper, familiar items also had a memory advantage independent of encoding time (500 or 1,000 ms). To conclude, with encoding times of 500 ms and above, working memory for characters seems no longer to depend on encoding time but, rather, on item familiarity. The cancelation of the familiarity effect for Pokémons under long study times may be specific to such material. Visual inspection of the Pokémons suggests that they are complex but perceptually more different than unfamiliar letters. Hence, with long encoding time, novices may also be able to represent unique features of Pokémons but not of characters that allow discrimination between items in working memory.

Another critical issue is the code used in working memory. We have claimed that we always observe effects of visual working memory, although Chinese literates have available phonological and semantic information on the characters. No doubt, people can recode items and use different codes (e.g., Lewis-Peacock et al., 2015). However, in a standard visual change detection task with hundreds of trials and short trial lengths, recoding seems not to be a strategy. The presence of articulatory suppression or of a verbal preload had no effect or only a marginal influence on the results of several experiments (Luck and Vogel, 1997; Morey and Cowan, 2004; Pelli et al., 2006; Sun et al., 2011; Xie and Zhang, 2017c). We observed that visual but not semantic similarity of foils impaired memory (Experiment 4). All of these results suggest a visual code. When verbal rehearsal was a likely strategy (Yu et al., 1985; Zhang and Simon, 1985), other results were observed. For example, if Chinese literates saw items sequentially presented at a slow pace (750 ms per item) and their memory was tested by a written recall, memory performance increased to seven characters, much higher than the “capacity” of VWM. If phonological recoding was rendered useless—the items were homophones or radicals without pronunciations—performance dropped to about three items, and the authors interpreted the smaller set of items as reflecting visual short-term memory. Hence, in change detection taks, even literates in Chinese should represent characters as visual information in working memory.

According to the sensory recruitment hypothesis of working memory, the visual representations should be represented in the same neural structures that encode the information. Therefore, for characters, activity should be seen in the FFC and the IPS, as these are involved in encoding and attentional control, respectively. The intensity of the neural signal should be a function of the necessary encoding and maintenance effort. In Experiment 5, therefore, trained material showed less neural activity than novel characters in the FFC and IPS. Activity increased with set size, approached a common maximum that was independent of training status, and lasted longer for higher set sizes. Whether the changed time courses are effects of encoding or maintenance or both cannot be decided. To test this, encoding and maintenance effort must be varied experimentally. Another result was that high performers generally showed more neural activity than low performers in the IPS, which we considered as task engagement. This conclusion also has to be substantiated by further empirical data.

In closing, some limitations of the brain imaging study need to be mentioned. We only compared neural activity in two regions for which we had specific hypotheses. However, working memory is provided by a network (Zimmer, 2008), which involves many regions that were not analyzed. Furthermore, we opted for a univariate approach in looking at the size of neural activity, not at its distribution (Postle, 2015). It is very likely that neural representations contribute to memory but are not expressed in elevated activity and are therefore not visible in a univariate approach. In a similar way, phonological or semantic information can also contribute to memory as part of a distributed network by making item representations distinct without having an influence that can be confirmed directly in elevated activity of an averaged signal or an overt behavioral measure. The phonology and meaning associated to a word form, for example, may stabilize a representation (Perfetti et al., 2013), even if these codes are not used to represent and address items in working memory. This might explain why word form training enhanced memory but did not allow the same memory performance as long experience with Chinese characters. Pelli et al. (2006) observed that after about 3,000 trials, Chinese illiterates reached the same efficiency in letter identification as experts but not the same memory performance. It was still one versus three items. In Experiment 5, after word form training, German participants remembered 1 novel and about 1.8 trained characters on average, whereas Chinese native speakers held 2.4 items in VWM. These results suggest that long experience causes a reorganization of character representations which enhances memory. Probably, it makes item representations more distinct and, in this way, reduces interference in working memory.

However, independent of these effects of prolonged practice, our experiments have shown that perceptual long-term knowledge makes an important contribution to the expertise advantage in VWM. We assume that experts can quickly assign perceived items within their field of expertise to a known category, allowing them unique representations in working memory. These categories assign even highly similar items perceptually to different tokens if they differ in at least one critical feature. These categories allow them to reject changed items if the change alters the category. In other words, due to the category change, even a perceptually small change becomes a big change that can be detected easily. We suggest that this mechanism provides the good memory performance and the seemingly high resolution that we also see in visual working memory experiments with experts in other fields of expertise.

## Data Availability Statement

The datasets generated for this study are available on request to the corresponding author.

## Ethics Statement

The studies involving human participants were reviewed and approved by the Ethics committee of Fakultät für Empirische Humanwissenschaften. The patients/participants provided their written informed consent to participate in this study.

## Author Contributions

HZ and BF designed the experiments and conducted all statistical analyses. BF collected the data of Experiment 2 and 5 and analyzed the brain imaging data. HZ mainly wrote the manuscript. Both authors edited the final draft and agreed on its content.

## Conflict of Interest

The authors declare that the research was conducted in the absence of any commercial or financial relationships that could be construed as a potential conflict of interest.

## Footnotes

1 Talairach coordinates in the left/right hemisphere.
