# Supplementary material for: Visual Working Memory of Chinese Characters and Expertise: The Expert’s Memory Advantage Is Based on Long-Term Knowledge of Visual Word Forms
Source: Front Psychol. 2020 Apr 17;11:516. doi: 10.3389/fpsyg.2020.00516 (PMC7180225; doi:10.3389/fpsyg.2020.00516)
Supplement: Supplementary file 1 [file Data_Sheet_1.PDF]

Supplementary Material

Chinese Characters and Expertise

1 General procedure

In Experiment 1 to 4, all participants were either Chinese native speakers (experts) or Germans without knowledge of Chinese language (novices). All studies had mixed designs with expertise as between subject factor; all other factors were varied within subjects. In Experiment 5, the training study, participants were German native speakers without knowledge of Chinese. Participants got course credit or a small lump sum as compensation for their participation. All behavioral experiments were run on computers controlled by E-Prime 2 (Psychology Software Tools, Pittsburgh). The trial structure matched the standard procedure of change detection tasks. A central fixation cue signaled presentation of the next study display. Each study display was shown for a short study time. The study display depicted a variable number (defining set size) of 'objects' (characters, figures, color patches). 'Objects' were drawn from a stimulus set without replacement so that no feature was repeated within a study display. In case of a non-match, one feature was replaced by a new one sampled from the stimuli that were left. After a short retention interval, the test display was presented and it remained visible until response. If not stated otherwise, a single 'object' was shown at one of the study locations. Participants were required to decide whether this item had been presented during study or something had changed. After an empty inter-trial interval, the next trial started.

Participants indicated their decision by a button press. The response buttons were two keys of the computer keyboard or two buttons on an external device (see below). No feedback was given. All factors except of expertise were varied within subjects and if not stated otherwise, all experimental conditions (change types, types of items, etc.) were fully randomized.

2 Material and Procedure of Experiment 1

2.1 Material

Table 1. Illustrations of the material used in Experiment 1.

傅	喉	渴	像	谨	谬	摊	湾
外	加	代	功	汉	仗	化	叹

0	255	157	44	76	240	255	179	102	45	250	0
0	244	221	183	17	107	0	28	0	99	152	0
255	0	39	190	96	38	0	144	0	49	191	0

Table 1 presents gray-scaled versions of characters used in Experiment 1 and printed versions of the used color patches. The numbers below the patches are the RGB values of the red, green, and blue color channel (in this order). Please note, because of the different color models, the printed colors give only an idea of their real hue on the monitor. In the experiment, the characters were presented in color ink, e.g. 傅 or 谬 in a size of about one visual degree on a 17" TFT with a resolution of 1280 ×1024.

2.2 Design & Procedure

The study was a 2 × 4 mixed design with the factors expertise (Chinese, German) and change type. Change type comprised three levels for characters: only the character changed, character's color changed, both features changed. In the fourth condition, color patches were presented. In each of the four conditions, we realized 28 no change trials and 28 change trials. The different trial types appeared in random order. The study displays depicted four items on four positions sampled from a pre-specified set of eight positions around the center of the screen at about 4° eccentricity. The characters were drawn from a set of 16 characters without replacement. Two of them were from the first row and two from the second row. The more complex ones had more strokes, than those in the second row. This was controlled but it was not analyzed as an experimental variable. Each 'object' of a study display was shown in a different color, which was selected from the set of 12 different colors. Within an array no color was repeated. One item of a study array was tested. Its position was randomly selected and it was in fifty percent matching and in fifty percent nonmatching.

Each trial started with a fixation cross (randomly varying between 400 to 600 ms), followed by the study display (500 ms). After an empty retention interval of 1000 ms, the test item was shown at its study location (self-paced, max time 2500 ms). Participants were required to decide whether the same item had been presented at that location or the probe had changed. The keys "c" and "m" on a standard keyboard were used as response keys.

3 Material and Procedure of Experiment 2

3.1 Material

Table 2. Illustration of the stimulus material in Experiment 2.

<table>
<tr><td colspan="3">像</td><td colspan="3">豹</td><td colspan="3">覆</td><td colspan="3">喉</td><td colspan="3">慎</td><td colspan="3">孰</td><td colspan="3">蛋</td><td colspan="3">蹄</td></tr>
<tr><td colspan="3">blue</td><td colspan="3">green</td><td colspan="3">red</td><td colspan="3">purple</td><td colspan="3">teal</td><td colspan="3">lime</td><td colspan="3">maroon</td><td colspan="3">cyan</td></tr>
<tr><td colspan="2">0
0
255</td><td colspan="2">255
244
0</td><td colspan="2">157
221
39</td><td colspan="2">44
183
190</td><td colspan="2">76
17
96</td><td colspan="2">240
107
38</td><td colspan="2">255
0
0</td><td colspan="2">179
28
144</td><td colspan="2">102
0
0</td><td colspan="2">45
99
49</td><td colspan="2">250
152
191</td><td colspan="2">0
0
0</td></tr>
<tr><td colspan="3">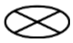</td><td colspan="3">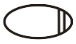</td><td colspan="3">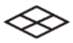</td><td colspan="3">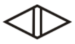</td><td colspan="3">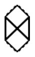</td><td colspan="3">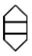</td><td colspan="3">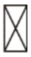</td><td colspan="3">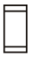</td></tr>
<tr><td colspan="3">喉</td><td colspan="3">喉</td><td colspan="3">喉</td><td colspan="3">喉</td><td colspan="3">喉</td><td colspan="3">喉</td><td colspan="3">喉</td><td colspan="3">喉</td></tr>
</table>

In the first row of Table 2, the used characters are shown. In this experiment, the pre-defined colors from E-Prime were used to define the colors in which the characters were presented. The colors were sampled from a list of eight without replacement. The E-Prime names of the colors used for characters are shown in the second row. The color patches (the numbers give the RGB values) were used in the color condition. The fourth row presents the eight figures used as geometric objects. The final row shows one character in the eight different fonts that were used. As fonts we selected (from left to right) four medium thick strokes (PMingLiU, HanWangYenLight, HanDing, HanWangShinSuMedium) and for bold strokes (HanWangCC02, HanWangZonYi, HanWangKaiBold, HanWangYenHeavy). Each study array displayed two characters in medium bold and two bold characters. If the font was changed the new font was taken from the same subset so that a font change did not change line thickness. Presentation was again on a 17” TFT.

3.2 Design and Procedure

Experiment 2 consisted of two 2 × 3 part designs with the factors expertise (Chinese, German) and change type. The six trial types of these two designs were mixed. One part design was realized with figures. This variation defines a 2 expertise (Chinese, German) × 3 change type (shape, pattern, color patch) mixed design. In the other part design, we presented characters realizing a 2 expertise (Chinese, German) × 3 change type (color, character, font) mixed design. All six change types of the two part designs were completely mixed and presented in random order.

Each trial started with a fixation cross presented in the center of the screen for 400 to 600 ms (randomly varying). Then the study array was visible for 500 ms showing four characters or

figures (in a size of about 1° vis degree) placed on randomly selected four positions out of a set of six positions. The positions were equidistant locations arranged on an invisible circle with a radius of 4° visual angle around the fixation cross. After 1000 ms retention interval a single test character (figure) was shown at its former position (at maximum for 2500 ms). Presentation was terminated by participants' responses (a key press).

In the figure conditions, four shapes or color patches were exposed. At test, one 'object' was presented at its study location and participants had to judge whether the 'object' had changed. The figures differed in their shape and inner pattern. The figures were constructed by combining four geometric shapes (ellipse, diamond, hexagon, rectangle) with different inner patterns (two crossing lines or two parallel lines). This resulted in eight figures (see the fourth row of the table). Always four of these figures were arranged to study arrays. The four 'objects' of a display were pseudo randomly selected so that on each array two of the 'objects' had the same outer feature, e.g. two ellipses and two diamonds were shown, and of each shape the two objects with different inner figures were used. The study arrays were all different combinations of figures that were possible with these restrictions. In match trials the same object was shown (62 trials). In big change trials, the test figure was changed in its shape (31 trials) and in small change trials, the inner pattern was changed (31 trials). In color trials, four color patches were presented during study (sampled from the list of 12 colors without replacement) and one color patch was shown at test, either in the same (34 items) or a new color (34 items).

In the character condition, we presented four Chinese characters in the same arrangement as the figures. The characters had different identities (word forms), each had another color and each was printed in another font. Each of these features could change but in a change trial only one feature was altered, the other two were repeated. All features were sampled from sets of eight without replacement. Ninety-six trials were no change trials (matching targets), 32 presented a changed character, 32 presented an old character in a new color, and 32 an old character in a new font.

4 Material and Procedure of Experiment 3

4.1 Material

The table presents the material used in Experiment 3. Only characters and their fonts were to be memorized. The two rows show them in the two used fonts. The fonts were “HanWangYenLight” and “PMingLiU”.

Table 3. Illustration of the stimulus material in Experiment 3.

像	豹	覆	喉	慎	孰	蛋	蹄
像	豹	覆	喉	慎	孰	蛋	蹄

4.2 Design & Procedure

The manipulation of the study test relation was blocked. Either one feature (character or font was relevant) or both had to be monitored. In the one feature condition, it was a 2 (German, Chinese) × 2 relevant feature (character, font) × 2 irrelevant feature (same, changed) design. In the both features condition, we had a 2 (German, Chinese) × 3 change type (character, font, both) design.

The blocks were presented in fixed order. First, a character block was realized in which only a character change was relevant, then a font block followed in which only font changes were relevant, and finally in the third block both features were relevant. Each one-feature block consisted of 36 trials, half of them were matching and half were nonmatching. If only one feature was relevant, half of the nonmatching trials changed also in the irrelevant feature and half did not. The both feature block was 42 items long. Twenty-one were matching trials and 21 nonmatching, one third in each of the possible non-match conditions. Each block was preceded by a practice block. All three blocks were then repeated in the same order without further practice blocks. Performances in the two repetitions were comparable and we therefore collapsed the data across repetitions.

The trial procedure was the same as in Experiment 2. However, we realized only a set size of three items. We sampled all memory sets from the list of eight characters without replacement. The fonts were randomly assigned to the characters in a way that each study array depicted two characters in one font and the other two in the alternative font.

5 Material and Procedure of Experiment 4

Table 4 presents the characters used in Experiment 4. They are arranged in three blocks of three rows separated by thick lines. The first block presents the characters used in the semantic relatedness condition, i.e. the semantic similarity between the study and test item varied. Items from the second block were used in the visual test manipulation, and the third block shows pseudo characters also varying in visual relatedness. Pseudo characters were constructed by rearranging radicals from different Chinese characters to new “characters” which do not exist in Chinese language. We would like to thank Prof. Fu, Institute of Psychology, Chinese Academy of Sciences, Beijing, for their assistance with item selection and the generation of pseudo characters.

Table 4. Illustration of the stimulus material of Experiment 2. Block 1 (row one to three) presents the characters in the semantic condition, block 2 (row four to six) are the characters used in the visual condition, and block 3 (row seven to nine) shows the constructed pseudo characters. The light-grey background is only used in table to indicate the row presenting the study items; in the experiment, the items were presented on white backgrounds.

看	常	闯	俺	逮	官	贫	缓	孤	堵
视	恒	仇	我	捕	吏	穷	慢	独	塞
高	基	咸	绘	哩	孙	复	忠	洗	昨
板	拌	捍	喝	唤	即	浆	拒	恳	形
版	伴	悍	渴	焕	既	奖	距	垦	刑
培	佶	肝	涛	逝	叮	栏	玲	牵	报
⿰足力	⿰王色	⿰礻九	⿰王甫	⿰子力	⿰纟奄	⿰忄先	仮	⿰月丁	哻
⿰足㕣	⿰扌色	⿰舌九	俌	⿰犭力	⿰忄奄	⿰犭先	版	⿰月丁	⿰氵旱
扐	⿰扌牙	⿰扌者	佸	⿰犭员	⿰忄者	⿰禾里	⿰片乍	⿰口尃	昄

Each block of the table consists of three rows. In the upper row (light-grey background), the character set is shown from which the study items were drawn. The middle and lower rows depict the characters of the corresponding non-match conditions. They either have a strong relatedness (middle row) or a weak relatedness (bottom row) to the study item shown in the first row of a block in the same column.

In the first, the semantic block, a highly related character was different in shape but it shared meaning with the study item (for translations see the examples in Figure 6 of the paper). The

items in the unrelated nonmatching condition (third row) had a different orthography and a different meaning.

In the second, the visual block, the highly related character shared a radical with the study item but it had a different meaning, again the unrelated item did not share features with the study item.

In the pseudo character condition (third block), the highly related pseudo characters shared a radical with the studied pseudo character, pseudo characters with a weak relatedness (third row) did not share radicals with the study items.

Pseudo characters were used to realize conditions with the same perceptual demands as characters but without unit representations in long-term memory. Hence, they should have no orthographic representation and neither a semantic nor a phonological representation. Problematic for this idea may be that pseudo characters are combinations of radicals and these sub-lexical units potentially can address long-term memory entries. However, it is theorized that perceptual identification happens at the level of characters as unit representations (Perfetti et al., 2005; Perfetti et al., 2013) and therefore long-term representations of radicals should have only minor relevance for working memory. In accordance with this, in a former study (Zimmer and Fu, 2008), Chinese native speakers showed much lower working memory performance for pseudo characters than for characters and their performance came close to the one of people who had no experience with Chinese language even though the radicals were known to Chinese native speakers. This supports that pseudo characters can be considered as novel character-like visual stimuli for Chinese literates. Additionally, in order to minimize possible influences of processing radicals on the results we introduced in this experiment an articulatory suppression task. At the beginning of each trial a number (randomly varying between 20 and 120) was presented for 1000 ms. Participants were required continuously to repeat the number aloud throughout the trial. They started uttering when the number was visible and stopped after responding.

5.1 Design & Procedure

In the character conditions, each study item presented four characters (in the same arrangement as in Experiment 1). Two of the characters were from the visual and two from the semantic character set. On the test display, a single test item was presented at its study location. In the pseudo character conditions four pseudo characters were shown. The test object was selected according to the similarity condition (same, high, low) to the item presented at this location in the study array. All conditions were mixed and the sequence of trial types was random. Participants were required to decide whether the target had changed or not. Considering the three sets of items and the three change types, this was a 3×3 within subject study design with 24 items in each of the two change conditions and 48 items in each match condition.

Timing was as following. A fixation cross was presented for 500 ms followed by presentation of the number for the articulatory suppression task (1000 ms). Then the study array was displayed for 200 ms and after a retention interval of 1000 ms the test item was shown until response. The next item followed after an inter stimulus interval of 2000 ms. Presentation time of the study array was in this experiment shorter than in previous experiments because only experts participated. The short presentation time should be sufficient for item encoding (Ngiam et al., 2019), but short enough to exclude strategic processing (Cowan, 2001). As before, responses were given on the computer keyboard.

6 Material and Procedure of Experiment 5

6.1 Material

This experiment was a training study in which participants acquired orthographic knowledge on a set of twelve characters and we tested their performance and neural activity before and after training. Two sets of characters were used. We selected 24 characters with four or five strokes and assigned them randomly to sets A and B of 12 characters each. Half of the participants practiced set A and set B were novel items in the post-test, the other half of participants practiced set B and set A was novel. In the analyses, we collapsed across this manipulation. Because the items were rear projected on a translucent screen in the MR experiment, the characters were shown in white on a grey background which provided the best visibility. Out of this reason they are depicted this way.

Set A:

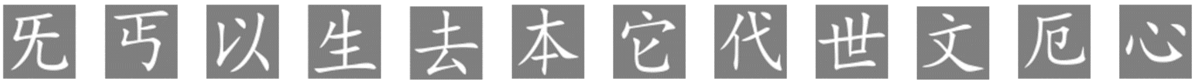

Set B:

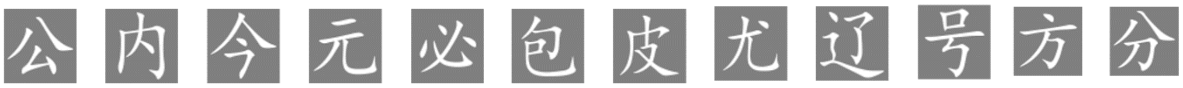

Figure 1. The items used in the training study.

6.2 The Change Detection Task

6.2.1 Design & Procedure

The benchmark for scoring practice effects was a *change detection task* with set sizes varying between one, two, and three items performed in a 3T scanner (Skyra, Siemens, Heidelberg, Germany). Participants responded by pushing buttons on MR compatible devices (NNL, Nordic Neuro Lab, Bergen, Norway) with their left and right index finger. Items were presented on a diffusing screen back-projected in a resolution of 1024 × 768. The size of the characters were about one visual degree. The screen was visible through mirrors mounted at the head coil. The trial parameters were as follows.

Each trial started with a fixation cross that was presented with an SOA between 300 and 700 ms to the study array (randomly sampled). The study display was shown for 1000 ms depicting one to three ‘objects’ around the center of the screen sampled from six positions as in Experiment 3. After a retention interval of 4000 ms, one character was presented at a central position as target for a maximum of 1500 ms, terminated by participant’s response. The characters were presented in white ink on a dark grey background. The inter-trial interval was jittered between 1000 and 6500 ms increasing in steps auf 500 ms. The frequencies of intervals across these time bins were specified according to a right-skewed distribution over increasing time following a geometric function.

The study realized a 3 × 2 design with the factors set size (1, 2, 3) and status (trained, novel). Participants saw 144 characters, 48 per set size. At posttest, half of the items were matches and half non-matches. Additionally, we presented one, two or three "random patches" as perceptual baseline (30 per set size) so that participants saw 234 items per session. These random patches were white squares of the same size as the characters filled with a random arrangement of black pixels which was generated by spatially randomizing the black pixels of the characters. These trials served as baseline in the MR analyses. The character-specific effects were estimated as contrasts between the character condition and the random patch condition per set size. The baseline trial structure was the same as for character trials. However, in these trials, participants had no memory task, they were required to watch the sequence of pictures and always to press the left (right) button when the single central stimulus appears. Response sides were balanced across participants.

6.2.2 MR parameters

We acquired T2-weighted functional images of 32 axial slices (3 mm thickness with .75 mm gap) in ascending order. The parameters were TR = 1800 ms, TE = 30 ms, flip angle = 90°, field of view 192 × 192 mm with a matrix size of 94 × 94. The 16th slice was used as mean functional image. Anatomy was measured by T1-weighted high resolution images (192 slices, .9 mm thickness) using MP-RAGE with TR =1900 ms, TE =2.13 ms, field of view 240 × 240 mm.

6.3 The Training Regime

Participants practiced the characters from their training set in 12 sessions (three per week). The training (outside the scanner) consisted of four tasks and it followed always the same scheme: stroke copying, character drawing, N-Back, free recall (from session 11 on). Each participant had three sessions per week. One week after the last training session the change detection task was administered in the scanner.

Stroke copying: For each character, participants saw videos which were screen shots of short animated writing sequences generated by the software eStroke (EON Media, Hong Kong) and presented via E-Prime. The video clips illustrated writing short stroke sequences. After each video clip, participants copied the presented writing sequence on a prepared sheet of paper (self-paced). The first clip showed only writing of the first stroke. Then in a second clip the first and second stroke were written and thereafter copied, and so on. After three or four such clips the complete character was written at once and participants wrote the complete character at once. This watch-copy sequence was repeated in each session with all characters of the training set.

Character drawing: Participants saw a character as static picture, it disappeared, and participants were required to write the character from memory. In order to increase the processing demands, over sessions, we shortened the presentation time from 4000 ms in session one to 1000 ms in the final session. The time provided for writing was self-paced. Participants started the next trial by a button press.

One-Back task: This task should practice reading. Participants saw 16 cycles of the 12 characters (defining a sequence of 192 items per cycle) randomly drawn from the trained

character set and reset after all characters were sampled. Participants task was spotting repetitions of items. They should push a button if the present item was the same as the previous one. The probability of an item repetition was .25. In the first session, presentation rate was slow. The character was presented for 1000 ms with an inter-stimulus interval of 1000 ms. Over the training sessions, presentation time was shortened. Every second session it was reduced by 200 ms so that in sessions 11 and 12 presentation time was 200 ms. In a parallel way, the inter-stimulus interval was increased to a variable length. In the final sessions, it was randomly sampled from the interval between 1000 and 4000 ms. This adaptive procedure increased the encoding demands as well as the memory demands over the 12 sessions.

Free recall: Starting with session seven, at the end of a training session, a free recall was administered. Participants should write down all practiced characters from memory without any external cue.

7 References

Cowan, N. (2001). The magical number 4 in short-term memory: A reconsideration of mental storage capacity. *Behav. Brain Sci.* 24(1), 87-185. doi: 10.1017/s0140525x01003922.

Ngiam, W.X.Q., Khaw, K.L.C., Holcombe, A.O., and Goodbourn, P.T. (2019). Visual working memory for letters varies with familiarity but not complexity. *J. Exp. Psychol. Learn. Mem. Cogn.* 45(10), 1761-1775. doi: 10.1037/xlm0000682

Perfetti, C., Cao, F., and Booth, J. (2013). Specialization and universals in the development of reading skill: How Chinese research informs a universal science of reading. *Sci. Stud. Read.* 17(1), 5-21. doi: 10.1080/10888438.2012.689786.

Perfetti, C.A., Liu, Y., and Tan, L.H. (2005). The lexical constituency model: Some implications of research on Chinese for general theories of reading. *Psychol. Rev.* 112(1), 43-59. doi: 10.1037/0033-295X.112.1.43.

Zimmer, H.D., and Fu, X. (2008). "Working memory capacity and culture-based expertise", in: *XXIV International Congress of Psychology.* (Berlin).
